# Trends in Incidence of Cancers of the Oral Cavity and Pharynx — United States 2007–2016

**DOI:** 10.15585/mmwr.mm6915a1

**Published:** 2020-04-17

**Authors:** Taylor D. Ellington, S. Jane Henley, Virginia Senkomago, Mary Elizabeth O’Neil, Reda J. Wilson, Simple Singh, Cheryll C. Thomas, Manxia Wu, Lisa C. Richardson

**Affiliations:** 1 Division of Cancer Prevention and Control, National Center for Chronic Disease Prevention and Health Promotion, CDC.

Cancers of the oral cavity and pharynx account for 3% of cancers diagnosed in the United States* each year. Cancers at these sites can differ anatomically and histologically and might have different causal factors, such as tobacco use, alcohol use, and infection with human papillomavirus (HPV) (*1*). Incidence of combined oral cavity and pharyngeal cancers declined during the 1980s but began to increase around 1999 (*2*,*3*). Because tobacco use has declined in the United States, accompanied by a decrease in incidence of many tobacco-related cancers, researchers have suggested that the increase in oral cavity and pharynx cancers might be attributed to anatomic sites with specific cell types in which HPV DNA is often found (*4*,*5*). U.S. Cancer Statistics^†^ data were analyzed to examine trends in incidence of cancers of the oral cavity and pharynx by anatomic site, sex, race/ethnicity, and age group. During 2007–2016, incidence rates increased for cancers of the oral cavity and pharynx combined, base of tongue, anterior tongue, gum, tonsil, oropharynx, and other oral cavity and pharynx. Incidence rates declined for cancers of the lip, floor of mouth, soft palate and uvula, hard palate, hypopharynx, and nasopharynx, and were stable for cancers of the cheek and other mouth and salivary gland. Ongoing implementation of proven population-based strategies to prevent tobacco use initiation, promote smoking cessation, reduce excessive alcohol use, and increase HPV vaccination rates might help prevent cancers of the oral cavity and pharynx.

Data on new cases of cancers of the oral cavity and pharynx (*International Classification of Diseases for Oncology, Third Edition:* C00–C14)^§^ reported during 2007–2016, the most recently available data, were obtained from U.S. Cancer Statistics. U.S. Cancer Statistics includes population-based cancer registry data from CDC’s National Program of Cancer Registries and the National Cancer Institute’s Surveillance, Epidemiology, and End Results (SEER) program. This report covers the entire U.S. population during the 10-year period. Only microscopically confirmed cases were included.

Annual incidence rates per 100,000 persons used modified annual population estimates in the denominator (as an approximation of person-years) and were age-adjusted by the direct method to the 2000 U.S. standard population^¶^ using SEERStat software (version 8.3.6; National Cancer Institute). Trends in rates were estimated using joinpoint regression, with a maximum of one joinpoint allowed (JoinPoint version 4.6.0; National Cancer Institute). Average annual percentage change (AAPC) for 2007–2016 was calculated using the average of the slope coefficients of the underlying joinpoint regression lines with the weights equal to the length of each segment over the interval. To determine whether AAPCs were significantly different from zero, a t-test was used for 0 joinpoints, and a z-test was used for 1 joinpoint. Rates were considered to increase or decrease if p<0.05. Rates were examined by anatomic site, sex, race/ethnicity (five mutually exclusive groups, including non-Hispanic white [white], non-Hispanic black [black], non-Hispanic American Indian/Alaska Native [AI/AN], non-Hispanic Asian/Pacific Islanders [A/PI], and Hispanic) and age group (20–39, 40–49, 50–59, 60–69, 70–79, and ≥80 years). Rates also were examined by association with HPV, based on studies that examined the presence of HPV DNA in a sample of cancer tissue specimens (*6*). HPV-associated cancers included squamous cell cancer types at the base of tongue, pharyngeal tonsils, anterior and posterior tonsillar pillars, glossotonsillar sulci, soft palate and uvula, and lateral and posterior pharyngeal walls. All other cancers were considered not HPV-associated.

During 2007–2016, incidence rates increased for cancers of the oral cavity and pharynx combined (0.6% per year on average), other oral cavity and pharynx (3.4%), base of tongue (1.8%), anterior tongue (1.8%), gum (1.9%), tonsil (2.4%), and oropharynx (1.9%) (Figure). Rates declined for cancers of the soft palate and uvula (−3.7%), hard palate (−0.9%), floor of mouth (−3.1%), lip (−2.7%), hypopharynx (−2.4%), and nasopharynx (−1.3%); and were stable for cancers of the cheek and other mouth and salivary gland. When cancers of the oral cavity and pharynx were grouped by association with HPV, HPV-associated cancers increased 2.1% per year on average, whereas cancers not associated with HPV decreased 0.4% per year on average.

**FIGURE:**
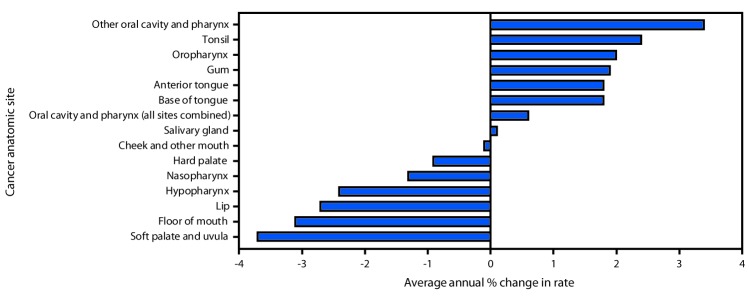
Trends in incidence of cancers of the oral cavity and pharynx,*^,†,§^ by cancer anatomic site, United States, 2007–2016 * Cancer incidence data were compiled from cancer registries that meet the data quality criteria for all invasive cancer sites combined, representing 100% of the U.S. population. ^†^ Annual percentage changes were statistically significant (at p<0.05) for all sites except “Salivary gland” and “Cheek and other mouth,” which had rates considered stable. ^§^ “Other oral cavity and pharynx” cancers included *International Classification of Diseases for Oncology, Third Edition* (ICD-O-3) codes C14.0 (Pharynx NOS), C14.2 (Waldeyers ring), and C14.8 (Overlapping lesion of lip, oral cavity, and pharynx).

Several anatomic sites are commonly grouped in the category “oral cavity and pharynx” (Table 1). Rates for all cancers of the oral cavity and pharynx combined increased among whites and A/PI, decreased among blacks and Hispanics, and were stable among AI/AN (Table 2). When the anatomic sites with increasing incidence trends were examined by race/ethnicity, rates increased only among whites with three exceptions: rates of cancers of the tonsil increased among AI/AN and of the anterior tongue and gum among A/PI. Rates of cancers of the base of the tongue, tonsil, and oropharynx decreased among blacks.

**TABLE 1:** Cancers of oral cavity and pharynx with ICD-O-3 code by anatomic site and HPV association status,* — United States, 2007 and 2016

Anatomic site	ICD-O-3 code	HPV-associated	No. of cases (%)	
			2007	2016
**Oral cavity and pharynx (all sites)**	**C00-C14**	**No**	**35,076 (100)**	**44,419 (100)**
Lip	C00.0-C00.9	No	2,048 (6)	1,847 (4)
Base of tongue	C01.9, C02.4, C02.8	Yes	5,661 (16)	8,164 (18)
Anterior tongue	C02.0-C02.3, C02.9	No	4,422 (13)	6,155 (14)
Floor of mouth	C04.0-C04.9	No	2,073 (6)	1,978 (4)
Gum	C03.0, C03.1, C03.9	No	1,215 (3)	1,727 (4)
Soft palate and uvula	C05.1, C05.2	Yes	870 (2)	743 (2)
Hard palate	C05.0, C05.8, C05.9	No	767 (2)	859 (2)
Cheek and other mouth	C06.0-C06.9	No	2,057 (6)	2,463 (6)
Salivary gland	C07.9-C08.9	No	3,862 (11)	4,433 (10)
Tonsil	C09.0-C09.9	Yes	5,791 (17)	8,792 (20)
Oropharynx	C10.0-C10.9	Yes	1,507 (4)	2,165 (5)
Nasopharynx	C11.0-C11.9	No	1,779 (5)	1,788 (4)
Hypopharynx	C12.9-C13.9	No	2,307 (7)	2,211 (5)
Other oral cavity and pharynx	C14.0-C14.8	Yes	717 (2)	1,094 (2)

**Abbreviations:** HPV = human papilloma virus; ICD-O-3 = *International Classification of Diseases for Oncology, Third Edition*.

* HPV-associated cancers are defined as cancers at specific anatomic sites with specific cell types in which HPV DNA frequently is found. These include ICD-O-3 site codes C01.9, C02.4, C02.8, C05.1, C05.2, C09.0, C09.8, C09.9, C10.0, C10.1, C10.2, C10.3, C10.4, C10.8, C10.9, C14.0, C14.2, and C14.8, with squamous cell carcinomas (histology codes: 8050–8084 and 8120–8131).

**TABLE 2:** Annual rate*^,†^ and average annual percentage change (AAPC) in rates of cancers of the oral cavity and pharynx, by trends, anatomic site, sex, race/ethnicity, and age group at diagnosis — United States, 2007–2016

Cancer type	Total cases 2007–2016	Year	Rate/AAPC	Sex		Race/ethnicity^§^					Age group (yrs)					
				Men	Women	NH White	NH Black	NH AI/AN	NH A/PI	Hispanic	20–39	40–49	50–59	60–69	70–79	≥80
**Cancer types with increasing trends**																
Oral cavity and pharynx (all sites)	400,291	2007	10.89	16.5	6.06	11.6	9.93	8.7	7.16	7.07	1.67	9.35	24.16	35.89	39.85	37.89
		2016	11.7	17.3	6.2	12.7	8.61	9.94	7.82	6.56	1.69	8.65	25.32	39.6	43.28	38.49
		(AAPC)	0.6^¶^	0.7^¶^	0.3	1.1^¶^	−1.5^¶^	1.7	0.9^¶^	−0.9^¶^	−0.5	−1.0^¶^	0.7^¶^	1.3^¶^	1.1^¶^	0.4
Base of tongue	69,460	2007	1.72	2.94	0.65	1.93	1.42	1.61	0.47	0.86	0.16	1.45	4.7	6.66	5.57	3.58
		2016	2.03	3.58	0.65	2.42	1.24	2.13	0.48	0.87	0.11	1.29	4.89	8.7	8.08	4.34
		(AAPC)	1.8^¶^	2.3^¶^	−0.5	2.5^¶^	−1.8^¶^	3.7	−0.3	−0.5	−4.4^¶^	−1.3^¶^	0.6	2.9^¶^	3.7^¶^	2.3^¶^
Anterior tongue	52,839	2007	1.39	1.76	1.05	1.55	0.66	─**	1.14	1.01	0.36	1.22	2.72	4.4	5.02	5.29
		2016	1.62	1.96	1.31	1.87	0.64	0.81	1.51	0.98	0.42	1.37	3.22	4.99	6.29	5.7
		(AAPC)	1.8^¶^	1.5^¶^	2.2^¶^	2.1^¶^	−0.4	─	2.9^¶^	0.3	0.8	0.8	2.1^¶^	1.9^¶^	2.8^¶^	0.9
Gum	14,583	2007	0.39	0.44	0.34	0.41	0.25	─	0.29	0.3	0.03	0.13	0.44	0.98	2.23	3.27
		2016	0.45	0.51	0.39	0.48	0.26	─	0.52	0.25	0.04	0.17	0.5	1.36	2.25	3.68
		(AAPC)	1.9^¶^	2.0^¶^	1.6^¶^	2.1^¶^	1.4	─	6.2^¶^	0.3	─	2.6	3.1^¶^	2.3^¶^	1.3	1.8^¶^
Tonsil	74,239	2007	1.76	2.98	0.64	1.96	1.6	1.15	0.43	0.98	0.17	2.31	5.92	5.55	3.88	1.79
		2016	2.22	3.88	0.7	2.62	1.53	1.88	0.58	1.23	0.14	2.26	7.12	8.33	5.83	2.62
		(AAPC)	2.4^¶^	2.7^¶^	1.4^¶^	3.4^¶^	−0.7^¶^	5.1^¶^	3.7	1.8	−3.4^¶^	−0.4	1.8^¶^	4.4^¶^	4.8^¶^	3.7^¶^
Oropharynx	18,010	2007	0.46	0.73	0.23	0.46	0.7	─	0.15	0.31	─	0.82	1.1	1.98	1.84	0.91
		2016	0.54	0.92	0.2	0.59	0.59	─	0.17	0.35	─	0.36	1.4	2.22	2.07	1.03
		(AAPC)	1.9^¶^	2.4^¶^	0.3	3.0^¶^	−2.3^¶^	─	−1.7	2.2	─	0.8	2.2^¶^	2.3^¶^	1.8^¶^	1.3
Other oral cavity and pharynx^††^	8,928	2007	0.22	0.36	0.1	0.22	0.3	─	─	0.23	─	0.14	0.47	0.83	0.93	0.85
		2016	0.28	0.46	0.11	0.3	0.27	─	─	0.19	─	0.15	0.58	1.07	1.18	1.08
		(AAPC)	3.4^¶^	3.8^¶^	2.0	4.6^¶^	−0.3	─	─	−1.6	─	1.9	4.0^¶^	3.5^¶^	3.3^¶^	3.4^¶^
**Cancers with decreasing or stable trends**																
Lip	20,180	2007	0.65	1.12	0.29	0.77	0.09	─	─	0.3	0.07	0.43	0.8	1.78	3.22	4.74
		2016	0.48	0.78	0.25	0.58	0.07	─	0.1	0.2	0.04	0.23	0.73	1.32	2.41	3.51
		(AAPC)	−2.7^¶^	−3.3^¶^	−1.4	−2.9^¶^	−3.2	─	─	−3.8^¶^	−4.3^¶^	−5.9^¶^	−0.4	−3.2	−2.7^¶^	−3.5
Floor of mouth	20,348	2007	0.64	0.98	0.34	0.68	0.63	0.98	0.27	0.4	0.03	0.55	1.47	2.49	2.46	1.7
		2016	0.5	0.7	0.31	0.56	0.39	0.61	0.18	0.23	0.03	0.24	1.19	1.88	2.12	1.66
		(AAPC)	−3.1^¶^	−3.5^¶^	−2.0^¶^	−2.3^¶^	−5.8^¶^	─	−2.3	−5.4^¶^	─	−7.9^¶^	−2.0^¶^	−3.0^¶^	−2.6^¶^	−2.0^¶^
Soft palate and uvula	8,158	2007	0.27	0.39	0.16	0.26	0.49	─	─	0.18	0.02	0.18	0.63	0.99	1.13	0.66
		2016	0.19	0.26	0.12	0.19	0.25	─	0.1	0.12	0.02	0.09	0.45	0.75	0.67	0.56
		(AAPC)	−3.7^¶^	−4.0^¶^	−3.2^¶^	−3.0^¶^	−6.0^¶^	─	─	−4.5^¶^	─	−6.7^¶^	−3.4^¶^	−3.4^¶^	−4.7^¶^	−1.8^¶^
Hard palate	8,308	2007	0.24	0.26	0.23	0.23	0.35	─	0.19	0.22	0.09	0.2	0.4	0.58	0.89	1.3
		2016	0.23	0.24	0.22	0.22	0.28	─	0.25	0.19	0.08	0.16	0.32	0.61	0.91	1.31
		(AAPC)	−0.9^¶^	−1.2	−0.4	−0.7	−1.9	─	−3.0^¶^	−4.0^¶^	−2.5^¶^	−3.2^¶^	−1.8	0.4	−1.0	0.3
Cheek and other mouth	22,559	2007	0.65	0.83	0.5	0.67	0.56	─	0.55	0.42	0.09	0.43	0.96	2.07	2.96	3.66
		2016	0.65	0.82	0.49	0.69	0.46	─	0.7	0.4	0.08	0.41	1.08	1.82	3.05	3.74
		(AAPC)	−0.1	0.1	−0.3	0.1	−2.4^¶^	─	2.8	−1.0	0.1	−1.7	1.2^¶^	−1.1^¶^	0.2	0.6
Salivary gland	42,238	2007	1.23	1.66	0.94	1.29	0.92	─	0.82	0.91	0.38	0.92	1.7	3.21	5.08	7.14
		2016	1.21	1.55	0.96	1.26	1.16	0.8	0.89	0.84	0.46	1.02	1.47	2.94	4.77	6.86
		(AAPC)	0.1	−0.4	0.6	0.1	2.3^¶^	─	1.4	−0.8	1.7^¶^	0.9	−0.5	−0.8^¶^	0.2	0.0
Nasopharynx	17,613	2007	0.56	0.82	0.32	0.44	0.69	─	2.3	0.45	0.24	0.69	1.28	1.42	1.43	1.05
		2016	0.49	0.73	0.26	0.34	0.71	0.62	1.91	0.34	0.2	0.65	1.1	1.4	1.17	0.69
		(AAPC)	−1.3^¶^	−1.3^¶^	−1. 4^¶^	−2.4^¶^	0.2	─	−1.7	−3.1^¶^	−1.7^¶^	−1.5	−1.3^¶^	−0.2	−2.4^¶^	−4.0
Hypopharynx	22,828	2007	0.71	1.24	0.27	0.68	1.27	0.89	0.3	0.5	─	0.38	1.56	2.95	3.22	2.06
		2016	0.55	0.94	0.21	0.55	0.76	0.6	0.38	0.37	0.03	0.22	1.27	2.21	2.47	1.72
		(AAPC)	−2.4^¶^	−3.2^¶^	−3.5^¶^	−1.9^¶^	−4.2^¶^	─	−1.5^¶^	−4.3^¶^	─	−5.6^¶^	−1.9^¶^	−2.4^¶^	−2.7^¶^	−0.9^¶^

**Abbreviations:** AI/AN = American Indian/Alaska Native; A/PI = Asian/Pacific Islander; NH = non-Hispanic.

* Per 100,000 standard population; overall rates were age-adjusted to the 2000 U.S. standard population.

^†^ Cancer incidence data were compiled from cancer registries that meet the data quality criteria for all invasive cancer sites combined, representing 100% of the U.S. population.

^§^ Racial and ethnic identifications are mutually exclusive. Hispanic persons can be any race. Rates are not presented for those with unknown or other race or unknown ethnicity.

^¶^ Significant at p = 0.05. Trends were measured with AAPC in rates and were considered to increase or decrease if p<0.05; otherwise rates were considered stable.

** Data suppressed for rates when the number of cases was <16 in a year.

^††^ “Other oral cavity and pharynx” cancers include *International Classification of Diseases for Oncology, Third Edition* (ICD-O-3) codes C14.0 (Pharynx NOS), C14.2 (Waldeyers ring), C14.8 (Overlapping lesion of lip, oral cavity and pharynx).

Rates for all cancers of the oral cavity and pharynx combined increased among males but were stable among females. Among females, rates increased for cancers of the anterior tongue, gum, and tonsil but decreased for cancers of the floor of mouth, soft palate and uvula, nasopharynx, and hypopharynx, and were stable for other sites. A similar pattern was observed among males except that rates also decreased for cancer of the lip, increased for cancers of the base of tongue, oropharynx, and other oral cavity and pharynx, and were stable only for hard palate, cheek and other mouth, and salivary gland.

By age group, rates for all cancers of the oral cavity and pharynx combined increased among persons aged 50–79 years, decreased among those aged 40–49 years, and were stable among those aged 20–39 and ≥80 years. Among sites with increasing rate trends, the increases were mainly driven by increases among persons aged 50–79 years; those rates were generally stable or decreased among persons aged 20–49 years and increased or were stable among those aged ≥80 years. All other rates were stable or could not be calculated because of small number of cases.

## Discussion

During 2007–2016, the incidence of cancers of the oral cavity and pharynx combined increased, despite decreases in several anatomic sites, including the nasopharynx, hypopharynx, lip, and floor of mouth. The overall increase appears to be driven by increases in cancers of the tonsil, base of tongue, oropharynx, and other cancers of the oral cavity and pharynx, which are HPV-associated, as well as by those of gum and anterior tongue.

Declines in tobacco use might have contributed to the decreases observed in some sites (*7*). Population-based tobacco control measures (including high-impact antitobacco mass media campaigns, tobacco price increases, and comprehensive smoke-free laws) are proven to prevent tobacco use initiation and promote smoking cessation,** but they are not implemented equally in all U.S. states and communities.^††^ Similarly, state alcohol control policies and alcohol screening are effective in reducing excessive alcohol use but are underutilized (*8*). Tobacco and alcohol use are still common in the United States; in 2018, 14% of the adult population reported current cigarette smoking, and 27% reported binge drinking.^§§^ To reduce the risk for cancers of the oral cavity and pharynx, communities might benefit from broader application of evidence-based interventions and targeted efforts among groups with high prevalence of tobacco and alcohol use or high cancer rates.^¶¶^

The overall increasing trend in oral cancer rates was the result of a combination of increasing rates among whites and A/PI, stable rates in AI/AN, and decreasing rates among blacks and Hispanics. A previous study found rates of oropharyngeal squamous cell cancers increased the most among white men compared with other racial/ethnic groups (*4*). Differences in sexual behavior might account for the higher rate; compared with other racial/ethnic groups, white men report an earlier age at oral sex initiation and have a higher number of oral sex partners which have been shown to be risk factors for exposure to HPV infection (*9*).

Public health efforts that focus on increasing HPV vaccination*** are an essential component of cancer prevention. Routine HPV vaccination is recommended for all persons at age 11 or 12 years, with catch-up vaccination through age 26 years.^†††^ CDC’s National Comprehensive Cancer Control Program supports cancer prevention efforts in all 50 states, the District of Columbia, tribal organizations, and U.S. territories; and, in collaboration with CDC’s National Center for Immunization and Respiratory Diseases (NCIRD), supports activities to promote and provide access to HPV vaccine. CDC’s Division of Cancer Prevention and NCIRD currently fund the American Cancer Society to convene partners at the National HPV Vaccination Roundtable to support activities that increase HPV vaccination coverage.^§§§^ There are no data on efficacy of vaccination on oral cavity and pharyngeal cancers from clinical trials, but these cancers are caused by HPV types that are targeted by available vaccines (*10*).

The findings in this report are subject to at least three limitations. First, delays in cancer reporting might result in an underestimate of incidence. Second, cancer registries do not routinely collect or report information about risk factors such as HPV infection, tobacco use, or alcohol use, so it was not possible to determine whether cancers occurred in persons exposed to these risk factors. Finally, because of the complexity of this anatomic region and potential difficulty in determining precisely where cancer originated, the anatomic site for some cases might have been incorrectly classified.

Cancers of the oral cavity and pharynx can be caused by exposure to risk factors that are common in the United States, including tobacco use, alcohol use, and HPV infection. Cancer control initiatives that use proven population-based strategies to prevent tobacco use initiation, promote smoking cessation, reduce alcohol use, and increase HPV vaccination rates could help reduce cancer risk.

**Summary**

**What is already known about this topic?**

Oral cavity and pharynx cancers account for 3% of cancers diagnosed annually in the United States; risk factors include tobacco use, excessive alcohol consumption, and HPV infection.

**What is added by this report?**

During 2007–2016, incidence of cancers of the oral cavity and pharynx combined increased, despite decreases in those at multiple anatomic sites. The overall increase was driven by increases in HPV-associated cancers of the tonsil, base of tongue, oropharynx, other oral cavity and pharynx, and the gum and anterior tongue.

**What are the implications for public health practice?**

Broader application of proven strategies to prevent tobacco use initiation, promote smoking cessation, reduce excessive alcohol consumption, and increase HPV vaccination rates can help reduce the incidence of these cancers.

## Footnotes

*
https://www.cdc.gov/cancer/dataviz .

†
https://www.cdc.gov/uscs .

§ http://www.iacr.com.fr/index.php?option = com_content&view = category&layout = blog&id = 100&Itemid = 577.

¶
https://seer.cancer.gov/popdata .

**
https://www.cdc.gov/tobacco/stateandcommunity/best_practices/index.htm?source = govdelivery

††
https://www.cdc.gov/statesystem/ .

§§
https://www.healthypeople.gov/2020/topics-objectives .

¶¶ https://www.thecommunityguide.org/topic/excessive-alcohol-consumption; https://www.thecommunityguide.org/topic/tobacco.

***
https://www.cdc.gov/vaccines/vpd/hpv/hcp/recommendations.html .

††† https://www.cdc.gov/cancer/ncccp/index.htm; https://www.cdc.gov/hpv/partners/index.html; https://www.thecommunityguide.org/topic/vaccination.

§§§
https://hpvroundtable.org/ .
